# Fecal immunochemical test surveillance in colorectal cancer following adenoma resection: A longitudinal, population-based linked cohort study in China

**DOI:** 10.1371/journal.pmed.1004687

**Published:** 2025-09-02

**Authors:** Jianhui Zhao, Chengcheng Liu, Jinhua Yang, Yanqin Huang, Liying Xu, Qian Xiao, Mingjuan Jin, Xiangxing Kong, Yeting Hu, Qilong Li, Suzhan Zhang, Xue Li, Shu Zheng, Kun Chen, Kefeng Ding

**Affiliations:** 1 Department of Colorectal Surgery and Oncology, Key Laboratory of Cancer Prevention and Intervention, Ministry of Education, The Second Affiliated Hospital, Zhejiang University School of Medicine, Hangzhou, China; 2 School of Public Health, The Second Affiliated Hospital, Zhejiang University School of Medicine, Hangzhou, China; 3 Zhejiang Provincial Clinical Research Center for CANCER, Cancer Center of Zhejiang University, Hangzhou, China; 4 Jiashan Institute of Cancer Prevention and Control, Jiashan, China; 5 Cancer Institute (Key Laboratory of Cancer Prevention and Intervention, China National Ministry of Education, Key Laboratory of Molecular Biology in Medical Sciences, Zhejiang Province), The Second Affiliated Hospital, Zhejiang University School of Medicine, Hangzhou, China; 6 Center for Medical Research and Innovation in Digestive System Tumors, Ministry of Education, Hangzhou, China; Vanderbilt University School of Medicine, UNITED STATES OF AMERICA

## Abstract

**Background:**

Although the fecal immunochemical test (FIT) is widely utilized in colorectal cancer (CRC) screening because of its noninvasive, rapid, and cost-effective characteristics, its effectiveness in post-adenoma resection surveillance remains unclear. This study aims to evaluate the benefits of follow-up FIT surveillance in individuals with adenoma resection and to identify risk factors associated with adenoma recurrence.

**Methods and findings:**

As part of China’s National Screening Project, we identified a total of 5,911 individuals who underwent adenoma removal during the first round of CRC screening in Jiashan and Haining between 2006 and 2021. All individuals with adenoma removal were invited to participate in a second CRC screening; 2,448 accepted and chose either direct colonoscopy surveillance (*n* = 989) or FIT surveillance (*n* = 1,459), while 3,463 declined. The Clone-Censor-Weight method was applied to mitigate time-related biases. Cox proportional hazards and Poisson regression models were used to evaluate the benefits of follow-up surveillance strategies after adenoma resection, adjusting for age, sex, baseline adenoma grade, family history of CRC in first-degree relatives, symptoms, chronic appendicitis or cholecystitis, and stressful life events. Additionally, we examined the risk factors associated with adenoma recurrence using logistic regression. The outcomes were the long-term incidence of CRC and the recurrence of adenomas following adenoma resection. Over average follow-up of 7.79 and 7.46 years, participants who underwent protocol-adherent follow-up FIT surveillance had a 44% lower CRC risk (hazard ratio [HR] = 0.56, 95% confidence interval [CI]: 0.31, 0.98; *p* = 0.044), and those who underwent direct colonoscopy had a 51% lower risk (HR = 0.49, 95% CI [0.27, 0.89]; *p* = 0.019), compared to individuals who refused follow-up surveillance. Compared with the direct follow-up colonoscopy group (53.56 per 100,000 person-years), the long-term CRC incidence rates were 70.38 for the follow-up negative FIT group and 80.14 for the positive FIT with adherence to colonoscopy group, with no statistically significant differences (*p* = 0.852; *p* = 0.834). Notably, participants who did not undergo colonoscopy following a positive FIT had a significantly increased CRC risk compared to those in the direct follow-up colonoscopy group, with an adjusted incidence rate ratio (aIRR) of 6.64 (95% CI [1.11, 39.83]; *p* = 0.038). Alcohol consumption (nondrinkers versus >3 times per week: adjusted odds ratio [aOR] = 0.43, 95% CI [0.27, 0.69]; *p* < 0.001) was associated with adenoma recurrence. Moreover, smoking (current smokers versus nonsmokers: aOR = 3.72, 95% CI [1.19, 11.60]; *p* = 0.024), obesity (obese versus normal: aOR = 3.21, 95% CI [1.17, 8.80]; *p* = 0.023), and having advanced adenomas at baseline (aOR = 3.30, 95% CI [1.41, 7.69]; *p* = 0.006) were associated with recurrence of advanced adenomas. Given the limited number of incident CRC cases and the observational study design, conclusions regarding the impact of follow-up FIT surveillance after adenoma removal should be interpreted with caution.

**Conclusion:**

Protocol-adherent follow-up FIT surveillance after adenoma removal was associated with reduced long-term CRC risk, comparable to that observed with direct colonoscopy. However, improving adherence to colonoscopy after a positive FIT surveillance is crucial.

## Author summary

**Why was this study done?:**

- Current guidelines in both China and the United States recommend that individuals who have undergone adenoma resection should have colonoscopic surveillance.
- Due to limited colonoscopy resources, high complication risks, and low acceptance, individuals with adenoma resection need better surveillance strategies.
- Although the fecal immunochemical test (FIT) is widely utilized in colorectal cancer (CRC) screening because of its noninvasive, rapid, and cost-effective characteristics, its effectiveness in post-adenoma resection surveillance remains unclear.

**What did the researchers do and find?:**

- As part of CRC screening, individuals who had undergone adenoma removal were invited to choose between direct colonoscopy surveillance and FIT surveillance. Our goal was to evaluate the benefits of follow-up surveillance strategies after adenoma resection.
- We also aimed to identify risk factors for adenoma recurrence.
- Protocol-adherent follow-up FIT surveillance was associated with a reduced long-term CRC risk in individuals with adenoma resection.
- Alcohol consumption was associated with adenoma recurrence, whereas current smoking, obesity, and the grade of adenomas removed were associated with advanced adenoma recurrence.

**What do these findings mean?:**

- Complete follow-up FIT surveillance may serve as an alternative to direct colonoscopy surveillance for monitoring individuals after adenoma resection.
- Improving participants’ adherence to colonoscopy following a positive FIT is crucial to ensure that follow-up FIT surveillance yields benefits.
- Surveillance should be prioritized for high-risk populations for adenoma recurrence, including men, heavy drinkers, and individuals with obesity.
- Given the limited number of incident CRC cases and the observational study design, causal inferences regarding the impact of follow-up FIT surveillance after adenoma removal should be made with caution.

## Abstract 3

Jianhui Zhao, Chengcheng Liu, Jinhua Yang, Yanqin Huang and colleagues evaluate the benefits of follow-up FIT surveillance in individuals with adenoma resection and identify risk factors associated with adenoma recurrence.

## Introduction

Colorectal cancer (CRC) ranks as the third most prevalent cancer and is the second leading cause of cancer-related mortality worldwide [1,2]. Current evidence indicates that most CRCs arise from adenomas via a multistep carcinogenic process [3,4], and the adenomas removal can significantly reduce the incidence and mortality associated with CRC [5–7]. However, due to the high recurrence rate of adenomas and the elevated risk of subsequent CRC compared to the general population [8], regular surveillance is strongly recommended for patients who have undergone intestinal polypectomy [9].

Patients with adenomas removal are advised to undergo colonoscopy surveillance at set intervals to prevent subsequent cancer development [8]; however, these intervals, defined by expert opinion, yield a low pathology output while interval cancers—CRC diagnosed between scheduled surveillance colonoscopies—continue to occur (e.g., 1.8% CRC yield versus 0.6% interval cancers) [10,11]. Additionally, colonoscopy is associated with low acceptance due to its invasive feature, complicated bowel preparation, and the risk of some serious complications (e.g., bleeding, perforation, or death) [12]. The demand for colonoscopy surveillance is significant, with the number of follow-up colonoscopies carried out stretching healthcare resources [13]. Furthermore, the widespread implementation of CRC screening has exacerbated the demand for endoscopic examinations, thus giving grounds for finding alternative surveillance strategies [14].

Biomarker-based approaches offer critical advantages over traditional screening methods such as imaging and endoscopy [15]. Current screening strategies are diverse, with colonoscopy being the gold standard for CRC diagnosis [16]. In contrast, fecal immunochemical tests (FITs) based on stool samples, are characterized by their noninvasive nature, high acceptance by population, and accurate detection of CRC [17]. FIT has been widely used in CRC screening programs and is associated with a reduced risk of CRC incidence and mortality [18–20], making it a potential alternative for surveillance in patients who have undergone adenoma removal. Previous evidence indicated that, compared to colonoscopy surveillance, FIT can achieve higher sensitivity for CRC while significantly reducing costs and demonstrating greater acceptability [14,21]. However, there is a paucity of research on the application of FIT for CRC surveillance in patients undergoing adenoma resection, and the long-term implications of such surveillance on CRC risk in this population remain largely unassessed. Therefore, it is necessary to further clarify the actual benefits of post-adenomatectomy FIT surveillance and optimize follow-up surveillance strategies.

Furthermore, identifying modifiable lifestyle factors aids in the comprehensive management strategies for individuals undergoing adenoma resection, as the progression of colorectal neoplasms is affected by heterogeneity and lifestyle [22–24]. Individuals with varying risk factors exhibit differing risks of adenoma recurrence, necessitating the adoption of risk-stratified surveillance strategies. Investigating modifiable lifestyle factors influencing adenoma recurrence may not only aid in establishing risk stratification for surveillance intervals post-polypectomy but also facilitate the promotion of primary prevention strategies for individuals with adenoma removal [25,26]. However, current research on risk factors for adenoma recurrence predominantly emphasizes adenoma characteristics, overlooking the consideration of healthy lifestyle factors [27].

Herein, we utilized data from the China CRC Screening Program to evaluate the potential benefits of follow-up FIT surveillance in individuals undergoing adenoma resection and to preliminarily explore the importance of colonoscopy adherence following positive FIT results, as well as identify risk factors associated with adenoma recurrence. We aimed to expand the understanding of surveillance methods for the adenoma removal population, thereby providing evidence for the precise prevention and control of CRC in the Chinese population.

## Methods

### Study design and population

**Fig 1:**
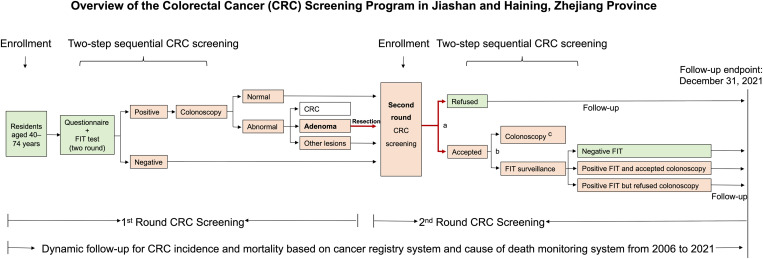
Overview of the colorectal cancer screening program in Jiashan and Haining, Zhejiang province. The red arrow represents the adenoma removal population included in this study. ^a^, all individuals who had adenomas removed will be invited for the second-round CRC screening, but they have the right to decline; ^b^, all individuals undergoing second-round CRC screening can choose either direct colonoscopy or sequential screening based on FIT surveillance; ^c^, these individuals underwent a colonoscopy without FIT surveillance. Abbreviation: CRC, Colorectal Cancer; FIT, fecal immunochemical test.

This study is part of China’s National Screening Project for the Early Diagnosis and Treatment of Common Cancers and serves as an ongoing retrospective cohort study focused on CRC screening. The project was initiated by the National Ministry of Health, starting in Jiashan County and Haining County of Zhejiang Province [28]. A two-step sequential CRC screening strategy was implemented for permanent residents in Jiashan and Haining aged 40–74 years. In step 1, individuals who accepted the screening invitation were interviewed using a questionnaire by trained interviewers and underwent two FIT at one-week intervals. A qualitative kit with a detection threshold of 100 ng hemoglobin/mL buffer or 20 µg hemoglobin/g feces (Abon Biopharm Co, Ltd) was used for the FIT. In the second step, those with at least one positive FIT were further invited to undergo a colonoscopy examination, which was conducted by trained endoscopic physicians at designated hospitals. Two rounds of repeated CRC screening were conducted from 2006 to 2021. Individuals not diagnosed with CRC—excluding cases detected during the first round of colonoscopy and interval cancers recorded by the cancer registry system—were invited to participate in the second round of screening. Additionally, those who declined the screening invitation in the first round were also invited to participate in the second round CRC screening (**Fig 1**).

**Fig 2:**
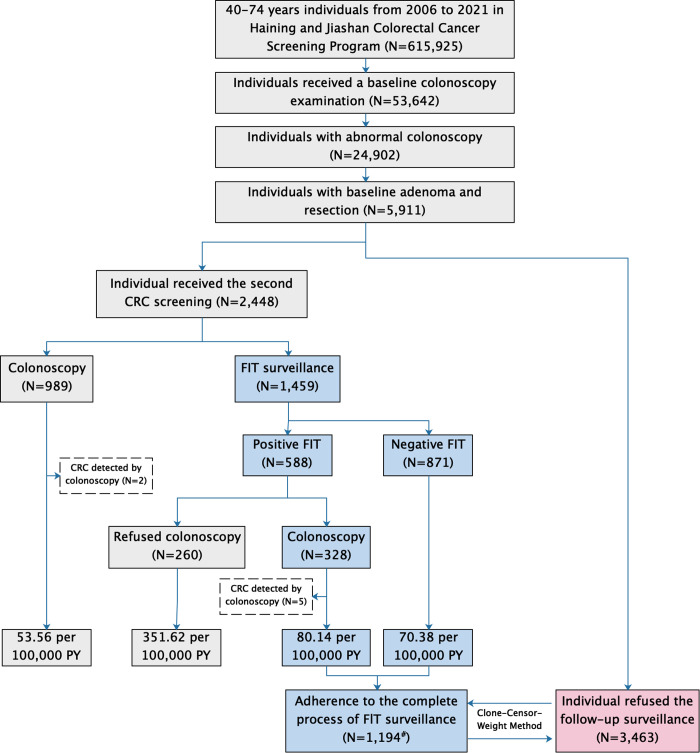
Flow diagram of included participants in the study. Abnormal colonoscopy findings included intestinal inflammation, bleeding, ulceration, adenomas, nonadenomatous polyps (such as hyperplastic, inflammatory, and serrated polyps), CRC, etc. ^#^, five CRC cases detected during colonoscopy were excluded. Abbreviations: FIT, fecal immunochemical test; CRC, colorectal cancer; PY, person-years.

This study was based on this two-round repeated CRC screening program in Jiashan and Haining cities, as illustrated in **Fig 2**. Specifically, a total of 615,925 individuals aged 40–74 who met the inclusion criteria were invited for CRC screening in Jiashan and Haining from 2006 to 2021, with 53,642 individuals undergoing colonoscopy. Among these, 24,902 individuals had abnormal colonoscopy results, and a total of 5,911 individuals who underwent adenoma removal were included. All individuals with adenoma removal were invited for second-round CRC screening, with 2,448 accepting and 3,463 declining. Among the 2,448 individuals who accepted the second-round CRC screening, participants could choose either direct colonoscopy or sequential screening based on FIT surveillance. Ultimately, 989 opted for direct colonoscopy, while 1,459 chose sequential screening based on FIT surveillance. We assessed the impact of adherence to the complete process of follow-up FIT surveillance and direct colonoscopy on reducing long-term CRC risk, compared to refusal of follow-up surveillance. Furthermore, we compared the long-term CRC risk among those who completed protocol-adherent follow-up FIT surveillance, those who underwent direct colonoscopy, and those who were nonadherent to colonoscopy after a positive FIT result. Additionally, based on the results of colonoscopy examinations from the second round of screening, we explored potential risk factors for adenoma and advanced adenoma recurrence.

A target trial emulation approach allows researchers to use observational data to replicate a ‘target’ randomized controlled trial (RCT)—that is, the RCT that would ideally be conducted if feasible—with exposures conceptualized as hypothetical interventions [29]. In this study, we emulated a target trial in which the intervention was defined as follow-up surveillance after adenoma removal, and the control group comprised individuals who refused follow-up surveillance (**Tables A and B in** S1 Appendix). This study is reported as per the Strengthening the Reporting of Observational Studies in Epidemiology (STROBE) guideline (S1 STROBE Checklist).

### Data collection

The screening dates, locations, colonoscopy findings, and FIT results of participants were extracted from the CRC screening program database. Histopathological information on removed lesions, including the size, number, location, and histology of adenomas and polyps, as well as diagnoses of CRC and other gastrointestinal issues (such as intestinal inflammation, bleeding, and ulceration), was assessed by pathologists and clinicians, and obtained from the medical systems of hospitals designated by the screening program. This data was matched with the CRC screening program database using a unique identifier. Demographic characteristics and CRC risk factors such as family history of CRC, clinical symptoms (e.g., constipation, diarrhea, mucous bloody stool), history of chronic appendicitis or cholecystitis, body mass index (BMI), alcohol consumption, smoking, and aspirin use, were gathered through face-to-face questionnaire surveys with participants.

### Outcomes and variates definition

The outcomes were the long-term incidence of CRC and the recurrence of adenomas following adenoma resection. CRC was defined according to International Classification of Diseases, 10th Revision (ICD-10) codes C18-C20. The final follow-up date extracted from the cancer registry and cause-of-death monitoring systems in this study was censored on December 31, 2021. The variates included in our analysis were age, sex, adenoma grade at baseline colonoscopy, family history of CRC in first-degree relatives, clinical symptom, chronic appendicitis or cholecystitis, stressful life events, BMI, smoking, alcohol consumption, aspirin use, colonoscopy interval, and adenoma grade. Specifically, BMI classification followed Chinese adult guidelines: <18.5 kg/m^2^ (underweight), 18.5 kg/m^2^ ≤ BMI < 24 kg/m^2^ (normal), 24 kg/m^2^ ≤ BMI < 28 kg/m^2^ (overweight), and≥ 28 kg/m^2^ (obese). Former and current smoking were defined as quitting for over 6 months and either consuming at least one cigarette daily for more than one year or over 300 cigarettes within 3 months, while alcohol consumption was defined as consuming ≥100 g of any alcohol per week for the past 6 months [30,31]. Clinical symptom was defined as individuals experiencing diarrhea, constipation, or bloody stool with mucus. Advanced adenoma refers to a conventional adenoma that is ≥10 mm in size or has advanced histology (high-grade dysplasia or villous/tubulovillous histology) [32].

### Statistical analysis

A description of the baseline characteristics was provided for both the complete follow-up FIT surveillance group and the nonfollow-up surveillance group. Categorical variables were presented as number (%). The follow-up time for CRC incidence began from the date of follow-up surveillance, extending until the earliest of the following events: diagnosis of CRC, death, or the end of follow-up on December 31, 2021. The long-term CRC incidence rates per person-year were calculated for different follow-up surveillance groups.

In this study, the Clone-Censor-Weight (CCW) method was implemented to deal with immortal time bias, using the *survivalCCW* R package [33]. The CCW method follows a three-step process [34,35]: (1) at the start of follow-up (the date of adenoma removal), each individual is cloned and assigned to each of the follow-up surveillance strategies under investigation; (2) these clones are monitored over time and artificially censored when their observed data diverges from the assigned surveillance strategy; and (3) the study population is reweighted to adjust for potential selection bias resulting from the artificial censoring. Inverse probability weighting Kaplan–Meier was used to compare cumulative CRC risks between the follow-up and nonfollow-up surveillance groups, adjusting for covariates including age, sex, adenoma grade at baseline colonoscopy, CRC family history in first-degree relatives, clinical symptom, chronic appendicitis or cholecystitis, and stressful life events. Specification and emulation of a target trial of CRC follow-up surveillance strategies are detailed in **Tables A and B in** S1 Appendix. Cox regression was used to examine the differences in incident CRC risk between the follow-up surveillance groups and the nonfollow-up surveillance group, estimating hazard ratio (HR) and their corresponding 95% confidence interval (CI).

Additionally, we employed a Poisson regression to calculate incidence rate ratios (IRRs), adjusted IRR (aIRR), and their corresponding 95% CI for CRC, with the nonfollow-up surveillance group serving as the reference. Model 1 aIRRs were derived from multivariable Poisson regression adjusted for age, sex, family history of CRC in first-degree relatives, and follow-up time. Model 2 included additional adjustments for clinical symptom, chronic appendicitis or cholecystitis, and stressful life events. Utilizing the Kaplan-Meier method with the log-rank test and Poisson regression, we compared the cumulative risk and incidence rates of CRC across three groups: the direct colonoscopy surveillance group, the protocol-adherent follow-up FIT surveillance group (comprising both FIT-negative individuals and those who adhered to colonoscopy after a positive FIT result), and the FIT-positive group who did not adhere to follow-up colonoscopy.

Additionally, we conducted univariate and multivariable logistic regression analyses to examine the associations between age, sex, alcohol consumption, aspirin use, smoking, BMI, colonoscopy interval, and adenoma grade at the first-round colonoscopy with adenoma and advanced adenoma recurrence, calculating odds ratios (ORs), adjusted OR (aOR), and 95% CI. In the multivariable analysis, variables with missing data were excluded.

The R software (version 4.1.2) was used to conduct statistical analyses employing a two-sided test and a significance threshold of 0.05.

### Ethics consideration

Ethical approval for the study was granted by the Committee on Research involving Human Subjects of the Second Affiliated Hospital of Zhejiang University (2017-072). The requirement for consent was waived by the Committee on Research involving Human Subjects of the Second Affiliated Hospital of Zhejiang University. The informed consent was waived because our retrospective study involved de-identified participant data and no more than minimal risk to the participants. Informed consent was obtained from all participants enrolled in China’s National Screening Project for the Early Diagnosis and Treatment of Common Cancers.

## Results

A total of 5,911 participants with adenoma removal were included in this study (**Fig 2**). 1,459 individuals received follow-up FIT surveillance. Of them, 1,199 completed the entire follow-up FIT surveillance process, including participants with a positive FIT who subsequently underwent colonoscopy and those with a negative FIT. After excluding 5 CRC cases detected during the referral colonoscopy, 1,194 individuals who completed the entire follow-up FIT surveillance were included in the CCW analysis. The results indicated that individuals who participated in complete follow-up FIT surveillance had a CRC incidence of 73.36 per 100,000 person-years. Additionally, 989 individuals underwent direct colonoscopy surveillance, while 3,463 did not participate in follow-up surveillance. Of the individuals with a positive follow-up FIT, 55.78% (328 out of 588) adhered to colonoscopy, resulting in the detection of 5 CRC cases during the referral colonoscopy. Among the 989 individuals who underwent direct colonoscopy, two cases of CRC were detected. The demographics of individuals in the FIT surveillance, direct colonoscopy, and refused surveillance groups at baseline colonoscopy are presented in **Table 1**.

**Table 1:** Demographics of the included population at baseline colonoscopy.

Characteristic	Total (*N* = 5,644)	Individual refused the surveillance (*N* = 3,463)	FIT surveillance^a^ (*N* = 1,194)	Direct colonoscopy (*N* = 987)	*P* value^b^
**Age, *n* (%)**					
<60	3,006 (53.26)	1,604 (46.32)	771 (64.57)	631 (63.93)	<0.001
≥60	2,638 (46.74)	1859 (53.68)	423 (35.43)	356 (36.07)	
**Sex, *n* (%)**					
Males	3,476 (61.59)	2,138 (61.74)	731 (61.22)	607 (61.50)	0.949
Females	2,168 (38.41)	1,325 (38.26)	463 (38.78)	380 (38.50)	
**Screening site, *n* (%)**					
Jiashan	3,602 (63.82)	2,324 (67.11)	841 (70.44)	437 (44.28)	<0.001
Haining	2042 (36.18)	1,139 (32.89)	353 (29.56)	550 (55.72)	
**Advanced adenoma, *n* (%)** ^c^					
No	4,750 (84.16)	2,985 (86.20)	959 (80.32)	806 (81.66)	<0.001
Yes	894 (15.84)	478 (13.80)	235 (19.68)	181 (18.34)	
**CRC family history in first-degree relatives, *n* (%)**					
No	4,958 (87.85)	3,039 (87.76)	1,042 (87.27)	877 (88.86)	0.512
Yes	686 (12.15)	424 (12.24)	152 (12.73)	110 (11.14)	
**Clinical symptom, *n* (%)** ^d^					
No	4,686 (83.03)	2,953 (85.27)	954 (79.90)	779 (78.93)	<0.001
Yes	958 (16.97)	510 (14.73)	240 (20.10)	208 (21.07)	
**Chronic appendicitis or cholecystitis, *n* (%)**					
No	4,097 (72.59)	2,517 (72.68)	863 (72.28)	717 (72.64)	0.963
Yes	1,547 (27.41)	946 (27.32)	331 (27.72)	270 (27.36)	
**Stressful life events, *n* (%)**					
No	5,568 (98.65)	3,425 (98.90)	1,167 (97.74)	976 (98.89)	0.008
Yes	76 (1.35)	38 (1.10)	27 (2.26)	11 (1.11)	

^a^FIT surveillance group refers to adherence to the complete process of FIT surveillance.

^b^*P* values were calculated based on comparisons of baseline demographic differences among the groups: Individual refused surveillance, FIT surveillance, and Direct colonoscopy.

^c^Advanced adenoma, any conventional adenoma ≥10 mm in size, or with advanced histology (high-grade dysplasia or villous/tubulovillous histology).

^d^Clinical symptom was defined as individuals experiencing diarrhea, constipation, or bloody stool with mucus.

Abbreviations: CRC, colorectal cancer; FIT, fecal immunochemical test.

**Fig 3:**
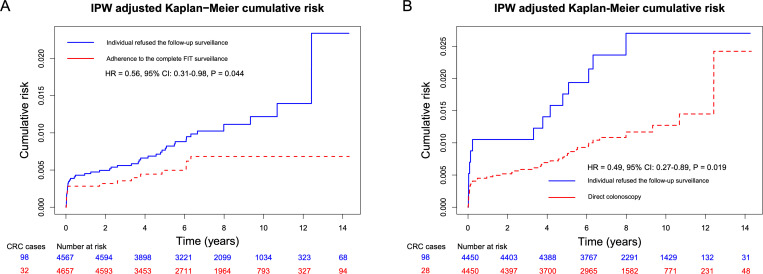
Comparison of CRC incidence between adherence to the complete process of follow-up FIT surveillance (A) and direct colonoscopy (B) vs. nonfollow-up surveillance groups among adenoma removal population based on the Clone-Censor-Weight method. HRs were calculated based on Cox regression. The covariates included in the Clone-Censor-Weight process were age, sex, adenoma grade at baseline colonoscopy, family history of CRC in first-degree relatives, symptoms, chronic appendicitis or cholecystitis, and stressful life events. Abbreviations: IPW, inverse probability weighting; FIT, fecal immunochemical test; CRC, colorectal cancer; HR, hazard ratio; CI, confidence interval.

**Fig 4:**
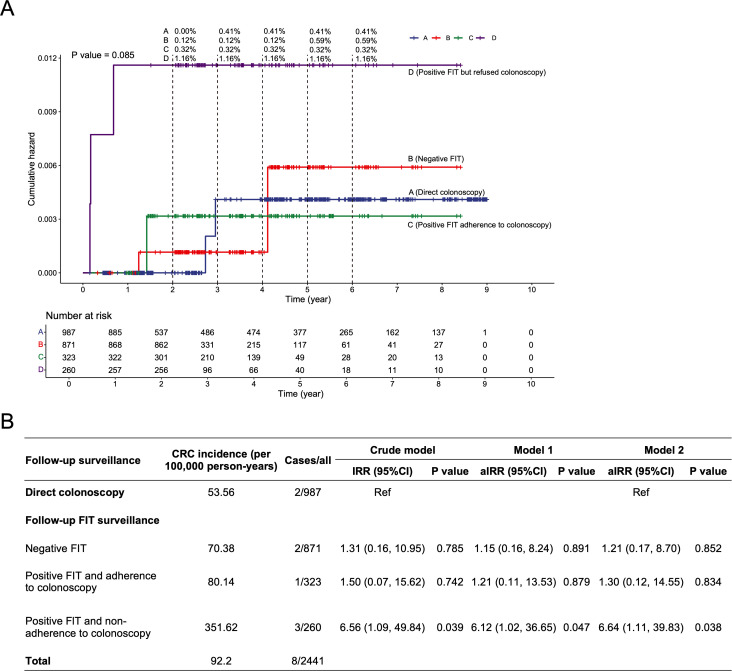
Comparison of CRC incidence among different follow-up surveillance groups following adenoma removal, utilizing Kaplan–Meier analysis (A) and Poisson regression (B). The Kaplan–Meier cumulative hazard estimates for CRC were unadjusted for covariates. The aIRR in Model 1 was estimated using multivariable Poisson regression, with adjustments for age, sex, family history of CRC in first-degree relatives, and accounting for follow-up time. Model 2 extended this adjustment by additionally controlling for clinical symptom, chronic appendicitis or cholecystitis, and stressful life events. The percentages above Fig 4A indicate the cumulative proportion of incident CRC cases at each time point. Abbreviations: FIT, fecal immunochemical test; CRC, colorectal cancer; IRR, incidence rate ratio; aIRR, adjusted incidence rate ratio; CI, confidence interval.

The results of CCW analysis indicated that participants who adhered to the complete follow-up FIT surveillance exhibited lower cumulative CRC risk compared to those who refused follow-up surveillance (HR = 0.56, 95% CI [0.31, 0.98]; *p* = 0.044) over average follow-up of 7.79 years (**Fig 3A**). Similarly, participants who adhered to the direct colonoscopy surveillance exhibited lower cumulative CRC risk compared to those who refused follow-up surveillance (HR = 0.49, 95% CI [0.27, 0.89]; *p* = 0.019) over average follow-up of 7.46 years (**Fig 3B**). Furthermore, in the different follow-up surveillance groups after adenoma removal, Kaplan-Meier curves showed that the CRC risk was higher in the group with a positive FIT who refused colonoscopy (**Fig 4A**). The results of the multivariable Poisson regression analysis indicated that individuals with a positive FIT who did not adhere to colonoscopy had a 6.64-fold increased risk of CRC (aIRR = 6.64, 95% CI [1.11, 39.83]; *p* = 0.038) compared to those who underwent direct colonoscopy surveillance (**Fig 4B**). Additionally, compared with the direct follow-up colonoscopy group (53.56 per 100,000 person-years), the long-term CRC incidence were 70.38 for the follow-up negative FIT group and 80.14 for the positive FIT with adherence to colonoscopy group, with no statistically significant differences (p for negative FIT = 0.852; p for positive FIT and adherence to colonoscopy = 0.834). Demographics of the direct colonoscopy group and the adherence to the complete process of FIT surveillance group at follow-up surveillance were presented in **Table C in** S1 Appendix.

In the second-round colonoscopy, 397 individuals were detected with adenomas (**Table 2**). Univariate analysis revealed that male sex was significantly associated with a higher risk of adenoma recurrence compared to female sex (aOR = 1.84, 95% CI [1.47, 2.31]; *p* < 0.001), whereas this association was not statistically significant in the multivariate analysis (aOR = 1.13, 95% CI [0.81, 1.58]; *p* = 0.456). In the univariate analysis, nondrinkers exhibited a 66% lower risk of adenoma recurrence (OR = 0.34, 95% CI [0.22, 0.52]; *p* < 0.001), with a 55% reduction observed in the multivariate model (aOR = 0.43, 95% CI [0.27, 0.69]; *p* < 0.001). Although current smoking was associated with an increased risk in the univariate model (OR = 1.61, 95% CI [1.31, 1.99]; *p* < 0.001), this association did not remain statistically significant after adjustment. Additionally, a longer colonoscopy interval was associated with an increased risk of adenoma recurrence in the univariate analysis; however, this association was not statistically significant in the multivariate model (aOR = 0.83, 95% CI [0.66, 1.04]; *p* = 0.105). In the univariate analysis, no association was observed between aspirin use, BMI, or advanced adenoma at the first-round colonoscopy and adenoma recurrence. For the recurrence of advanced adenomas, the multivariate model indicated current smoking (current smokers versus nonsmokers: aOR = 3.72, 95% CI [1.19, 11.60]; *p* = 0.024), obesity (obese versus normal: aOR = 3.21, 95% CI [1.17, 8.80]; *p* = 0.023), and having advanced adenomas at baseline (aOR = 3.30, 95% CI [1.41, 7.69]; *p* = 0.006) were significantly associated with recurrence (**Table D in** S1 Appendix), although the wide CIs warrant cautious interpretation.

**Table 2:** The risk factors associated with adenoma recurrence among the population following the removal of adenomas after the first-round colonoscopy.

Variable	Nonadenoma (*N* = 864)	Adenoma (*N* = 397)	*P* value	OR (95%CI)	*P* value	aOR (95%CI)^a^	*P* value
**Age, *n* (%)**							
<60	312 (36.5)	126 (32.0)	0.133	Ref			
≥60	542 (63.5)	268 (68.0)		1.15 (0.93, 1.42)	0.195	1.20 (0.94, 1.53)	0.136
**Sex, *n* (%)**							
Females	380 (44.0)	99 (24.9)	<0.001	Ref			
Males	484 (56.0)	298 (75.1)		1.84 (1.47, 2.31)	<0.001	1.13 (0.81, 1.58)	0.456
**Alcohol consumption** ^b^ **, *n* (%)**							
>3 times per week	118 (13.7)	132 (33.2)	<0.001	Ref			
1–2 times per week	10 (1.2)	8 (2.0)		0.84 (0.41, 1.72)	0.636	0.88 (0.42, 1.83)	0.727
1–2 times per month	252 (29.2)	158 (39.8)		0.73 (0.58, 0.92)	0.008	0.84 (0.64, 1.10)	0.199
Never	110 (12.7)	24 (6.0)		0.34 (0.22, 0.52)	<0.001	0.43 (0.27, 0.69)	<0.001
Unkown	374 (43.3)	75 (18.9)					
**Aspirin use, *n* (%)**							
Yes	29 (8.2)	14 (5.9)	0.363	Ref			
No	324 (91.8)	224 (94.1)		1.26 (0.73, 2.15)	0.409		
**Smoking** ^c^ **, *n* (%)**							
No	528 (61.1)	191 (48.1)	<0.001	Ref			
Current	216 (25.0)	162 (40.8)		1.61 (1.31, 1.99)	<0.001	1.32 (0.99, 1.76)	0.062
Former	18 (2.1)	12 (3.0)		1.51 (0.84, 2.7)	0.169	1.03 (0.55, 1.94)	0.922
Unkown	102 (11.8)	32 (8.1)					
**BMI** ^d^ **, *n* (%)**							
Normal	286 (33.1)	173 (43.6)	<0.001	Ref			
Underweight	24 (2.8)	9 (2.3)		0.72 (0.37, 1.41)	0.344		
Overweight	170 (19.7)	116 (29.2)		1.08 (0.85, 1.36)	0.541		
Obese	42 (4.9)	31 (7.8)		1.13 (0.77, 1.65)	0.541		
Unkown	342 (39.6)	68 (17.1)					
**Colonoscopy interval** ^e^ **, *n* (%)**							
<6 years	501 (58.0)	200 (50.4)	0.014	Ref			
≥6 years	363 (42.0)	197 (49.6)		1.23 (1.01, 1.5)	0.037	0.83 (0.66, 1.04)	0.105
**Advanced adenoma at first-round colonoscopy** ^f^ **, *n* (%)**							
No	688 (79.6)	331 (83.4)	0.136	Ref			
Yes	176 (20.4)	66 (16.6)		0.84 (0.64, 1.09)	0.195		

^a^ Variables with missing data were excluded from the analysis.

^b^ Alcohol drinking was defind as consuming ≥100 g of any alcohol per week over the past 6 months.

^c^ Former and current smoking were defined as quitting smoking for more than 6 months before colonoscopy and consuming at least one cigarette per day for more than one year or consuming over 300 cigarettes within 3 months, respectively.

^d^ BMI was classified according to the the guideline for Chinese adults: <18.5 kg/m^2^ (underweight), 18.5 kg/m^2^ ≤ BMI < 24 kg/m^2^ (normal), 24 kg/m^2^ ≤ BMI < 28 kg/m^2^ (overweight) and ≥ 28 kg/m^2^ (obese).

^e^ Colonoscopy interval refers to the time period between the first and second-round colonoscopy examinations.

^f^ Advanced adenoma, any conventional adenoma ≥10 mm in size, or with advanced histology (high-grade dysplasia or villous/tubulovillous histology).

Abbreviation: OR, odds ratio; aOR, adjusted odds ratio; CI, confidence interval; BMI, body mass index.

## Discussion

This was a population-based study in China demonstrating that complete follow-up FIT surveillance after adenoma resection was associated with a reduced long-term risk of CRC compared to no follow-up surveillance. More importantly, the incidence of CRC among participants who underwent direct follow-up colonoscopy was comparable to that among those who completed protocol-adherent FIT-based surveillance. However, the CRC risk among participants who refused colonoscopy following a positive FIT was 6.64 times higher than that of those who underwent direct follow-up colonoscopy surveillance. Notably, the limited number of CRC cases warrants cautious interpretation. Additionally, alcohol consumption was associated with adenoma recurrence, whereas current smoking, obesity, and the grade of adenomas at baseline colonoscopy were associated with advanced adenoma recurrence.

Early endoscopic resection of adenoma is an effective method to prevent the progression of cancer [36]. Clinical studies have confirmed that adenomas are prone to relapse, with a recurrence rate of up to 20%–50% [37–39]. And appropriate follow-up surveillance after polypectomy has important preventive significance. Available surveillance guidelines recommend colonoscopy-based risk-stratification strategies for individuals who have undergone adenoma removal. However, compliance with colonoscopy surveillance may be affected by anxiety over the preparation and procedure, including fears of bowel perforation, pain, and embarrassment [40]. In a Korean Association for the Study of Intestinal Diseases multicenter study, 60.1% of patients showed late adherence to surveillance colonoscopy after polypectomy [41]. In the general population, after FIT was implemented by Kaiser Permanente in Northern California (2000–2015), screening compliance rose from 38.9% to over 80%, accompanied by significant reductions in annual CRC incidence [19]. Direct colonoscopy-based surveillance after polypectomy places a significant burden on both patients and healthcare systems, whereas FIT-based surveillance, due to its noninvasive nature in detecting CRC and precursor lesions, effectively stratifies screening populations for colonoscopy, thereby reducing psychological strain on individuals and alleviating the endoscopy burden on healthcare resources [16]. FIT surveillance may reduce the incidence of CRC in populations, primarily due to its ability to detect not only CRC but also precursor lesions such as polyps, adenomas, and advanced adenomas [42]. This capacity may facilitate the early detection of CRC and contribute to the prevention of CRC in individuals who have undergone adenoma removal by identifying these precursor lesions.

Previous evidence has indicated that FIT has comparable accuracy in detecting colorectal neoplasia during surveillance of individuals with prior adenoma removal [43,44]. A study conducted in England reported that annual low-threshold FIT at a concentration of 40 µg/g, when combined with colonoscopy in positive cases, achieves high sensitivity for CRC and is likely to be cost-effective compared to conducting colonoscopy triennial colonoscopy [21]. Thus, the FIT could serve as a feasible option for surveillance, as participants demonstrate high acceptability due to its noninvasive nature and the requirement of only a stool sample. This is supported by a RCT conducted across six centers in China, which found participation rates of 42.5% (1,665 out of 3,916) for the colonoscopy group and 94.0% (7,386 out of 7,854) for the FIT group [45]. Notably, although FIT exhibits higher sensitivity for CRC, its ability to detect advanced adenomas is comparatively limited [21]. This suggests that improving FIT’s sensitivity for advanced adenomas or other CRC precursor lesions, while maintaining specificity, may be a key focus for the next steps in noninvasive CRC screening. Additionally, a positive FIT may be associated with a higher acceptance of colonoscopy due to psychological cues and an increased CRC detection rate during the procedure. Given the uncertainty regarding the benefits of follow-up FIT surveillance for individuals who have undergone adenoma resection in China, our findings provide evidence that protocol-adherent follow-up FIT surveillance was associated with a reduced risk of developing CRC.

Our study suggests that follow-up FIT surveillance enables risk stratification into FIT-negative and FIT-positive groups, whereby only the latter proceed to colonoscopy. As a triage tool, FIT helps identify individuals at higher risk for CRC, thereby improving the diagnostic yield of CRC and advanced lesions during colonoscopy. Early removal of these abnormal lesions can prevent CRC. On the other hand, FIT-negative individuals represent a low-risk group that does not need further colonoscopy. This comprehensive follow-up FIT monitoring allows the CRC incidence in both groups to remain comparable to that of direct colonoscopy screening, effectively conserving colonoscopy resources, improving adherence among adenoma resection patients, and reducing long-term CRC incidence. However, optimal surveillance strategies should be guided by a comprehensive assessment of long-term reductions in CRC incidence or mortality, potential harms (e.g., colonoscopy-related complications or psychological distress), and associated costs for both the healthcare system and patients. Compared to direct colonoscopy, sequential surveillance based on FIT offers a resource-efficient and widely acceptable alternative. Furthermore, our findings suggested that individuals who have undergone adenoma resection and have a positive FIT surveillance but do not adhere to colonoscopy face over a 7-fold increased CRC incidence compared to those who undergo direct colonoscopy surveillance. Therefore, efforts should focus on improving adherence to colonoscopy after a positive FIT result to ensure that follow-up FIT surveillance produces its maximum benefit in the adenoma resection population.

Our previous research found that individuals who underwent adenoma removal at baseline colonoscopy may have an elevated long-term risk of CRC compared to the general population or individuals without adenomas [46]. Given that current guidelines recommend colonoscopy surveillance for individuals with prior adenoma removal, the large number of such individuals imposes a substantial burden on colonoscopy resources [47]. Therefore, initiating with primary prevention and identifying risk factors for adenoma recurrence may represent a cost-effective approach. Early prevention based on risk stratification holds substantial potential for reducing the burden on colonoscopy resources [48]. As previously reported [49], our finding indicated that the recurrence risk of colorectal adenomas was associated with the gender of the patient, with sex males having a higher risk of recurrence compared to sex females. It is recommended that male individuals remain vigilant and actively engage in surveillance measures to prevent the recurrence of adenomas [50]. Our study found that never drinking alcohol reduces the risk of adenoma recurrence by 55%. Consistent with recent findings from a study conducted in China, alcohol consumption is identified as an independent risk factor for the recurrence of adenomas [51]. A meta-analysis exploring the relationship between alcohol consumption and adenoma risk demonstrated a positive dose-response association [52]. Therefore, the aforementioned evidence consistently suggests that individuals in the adenoma resection population should reduce their alcohol consumption.

Additionally, we observed that obesity was associated with the recurrence of advanced adenomas. In line with the findings of Shen and colleagues [53], individuals with obesity exhibited a significantly elevated risk of advanced adenomas compared to those with a normal weight. Jacobs and colleagues reported that the odds of adenoma recurrence in men was elevated in overweight patients and obese patients over a median follow-up of 3.1 years among 2,465 patients [54]. They also demonstrated that a high BMI was a slightly stronger risk factor for advanced adenoma recurrence when compared with nonadvanced lesions. A Korean study involving 193 participants also reported that, during a median follow-up period of 4.8 person-years, a high BMI (≥25 kg/m^2^) was associated with the recurrence of advanced adenomas, but not with the overall recurrence of adenomas [55]. The findings of Jung and colleagues also align with this, indicating that a higher BMI has a more pronounced impact on the advanced adenoma recurrence than on any adenoma recurrence [56]. Thus, weight management could be a strategy to reduce the risk of adenoma recurrence.

This study has several limitations that should be acknowledged. First, while examining the impact of follow-up surveillance on CRC incidence, the small number of CRC cases led to wide CIs for some estimates and limited the power of subgroup analyses. Therefore, the findings in the study should be interpreted with caution. Second, although we adjusted for available confounding factors, potential residual confounding may still exist because information on demographic characteristics and other factors related to CRC risk was limited in the data collected for this study. Third, the questionnaire regarding lifestyle was collected at follow-up FIT surveillance, and it may not fully capture changes in lifestyle over the surveillance interval. Thus, there might be some confounding in determining the relationship between related factors and the risk of adenoma recurrence. Fourth, 3,463 individuals declined the invitation for follow-up surveillance, and the specific reasons for their refusal were not thoroughly documented for analysis. Understanding these reasons could provide valuable insights for improving participation in FIT surveillance in the future. Additionally, the lack of comprehensive records for participants who underwent other screening or surveillance procedures during the study period may introduce potential outcome bias. However, a small follow-up survey indicated that less than 5% of individuals underwent a paid colonoscopy at hospitals outside the screening program [30]. Finally, although we conducted an emulated trial using real-world data based on the CCW method, the observational design limits our ability to make causal inferences about the impact of follow-up FIT surveillance on CRC risk in individuals who have undergone adenoma removal. High-quality evidence from future RCTs is needed to confirm the benefits of follow-up FIT surveillance in preventing CRC in this population.

In conclusion, protocol-adherent follow-up FIT surveillance was associated with reduced long-term CRC incidence after adenoma resection, showing comparable effectiveness to direct colonoscopy surveillance. However, nonadherence to colonoscopy following a positive FIT result may negate these benefits. Surveillance should be prioritized for populations at high risk of adenoma recurrence, including heavy alcohol consumers, current smokers, and individuals with obesity. Further research is needed based on RCTs to demonstrate the benefits, frequency, and optimal intervals of follow-up FIT surveillance.

## Supporting information

S1 STROBE Checklist STROBE Statement—checklist of items that should be included in reports of observational studies. This checklist is licensed under the Creative Commons Attribution 4.0 International License (CC BY 4.0; https://creativecommons.org/licenses/by/4.0/).
(DOC)

S1 Appendix Supplementary tables. **Table A.** Specification and emulation of a target trial for CRC with complete follow-up FIT surveillance and nonfollow-up surveillance strategies. **Table B.** Specification and emulation of a target trial for CRC follow-up with direct colonoscopy and nonfollow-up surveillance strategies. **Table C.** Demographics of the population at the second round of CRC screening. **Table D.** The risk factors associated with advanced adenoma recurrence among the population following the removal of adenoma after the first-round colonoscopy.
(DOCX)

## Abbreviations

aIRR: adjusted incidence rate ratio
aOR: adjusted odds ratio
BMI: body mass index
CCW: Clone-Censor-Weight
CI: confidence interval
CRC: colorectal cancer
FIT: fecal immunochemical test
HR: hazard ratio
ICD-10: International Classification of Diseases, 10th Revision
IRR: incidence rate ratio
OR: odds ratio
RCT: randomized controlled trial
STROBE: Strengthening the Reporting of Observational Studies in Epidemiology
