# Supplementary material for: Fecal immunochemical test surveillance in colorectal cancer following adenoma resection: A longitudinal, population-based linked cohort study in China
Source: PLoS Med. 2025 Sep 2;22(9):e1004687. doi: 10.1371/journal.pmed.1004687 (PMC12416832; doi:10.1371/journal.pmed.1004687)
Supplement: S1 Appendix — Table A. Specification and emulation of a target trial for CRC with complete follow-up FIT surveillance and nonfollow-up surveillance strategies. Table B. Specification and emulation of a target trial for CRC follow-up with direct colonoscopy and nonfollow-up surveillance strategies. Table C. Demographics of the population at the second round of CRC screening. Table D. The risk factors associated with advanced adenoma recurrence among the population following the removal of adenoma after the first-round colonoscopy. (DOCX) [file pmed.1004687.s002.docx]

**Supplementary tables**

**Fecal Immunochemical Test Surveillance in Colorectal Cancer Following Adenoma Resection: A longitudinal, population-based linked cohort study in China**

J Zhao et al.

**Table A.** Specification and emulation of a target trial for CRC with complete follow-up FIT surveillance and non-follow-up surveillance strategies.

| **Component** | **Target trial** | **Emulated trial using RWD** |
| --- | --- | --- |
| Design | Open-label two-parallelarm superlority randomized trial | Cohort study |
| Aim | Estimate the effect of receiving complete follow-up FIT surveillance on the long-term CRC risk in individuals who have undergone adenoma removal | Same |
| Inclusion | Individuals with adenoma removal at baseline colonoscopy | Same |
| Exclusion | Individuals who are non-compliant with the complete process of follow-up FIT surveillance (positive FIT but refuse colonoscopy); CRC detected by colonoscopy | Same |
| Treatment strategies | 1. Follow-up FIT surveillance within 9.2 years after adenoma removal 2. Refusing follow-up surveillance within 9.2 years after adenoma removal | Same |
| Treatment assignment | Patients are randomly assigned to either strategy | Patients are non-randomly assigned to a follow-up surveillance strategy. Randomization is emulated via cloning of patients in both arms |
| Treatment implementation | None | 9.2 years grace period |
| Outcome | Incident CRC after surveillance strategies assignment | Same |
| Type of outcome | Time of CRC development | Same |
| Follow up | Follow up starts at adenoma removal, equivalent to surveillance assignment | Follow up starts at adenoma removal, which does not correspond to surveillance assignment |
| Censoring | Loss to follow up, administrative censoring, death | Loss to follow up, administrative censoring, death |
| Adjustment variables | Age, sex, adenoma grade at baseline colonoscopy, CRC family history in first-degree relatives, clinical symptoms, chronic appendicitis or cholecystitis, stressful life events | Same |
| Causal contrast andanalysis plan | Intention-to-treat and per-protocol effect analysis: Excluding non-compliant individuals with the complete process of follow-up FIT surveillance. A weighted Kaplan-Meier estimator and Cox regression were used to compute 14.4-year risk differences and hazard ratio | Per protocol effect only. Excluding non-compliant individuals with the complete process of follow-up FIT surveillance. Analysis in each arm of the cloned data, a weighted Kaplan Meier estimator and Cox regression were used to compute 14.4-year risk differences and hazard ratio |
| Estimands | Differences in incident CRC rate and hazard ratio between the follow-up FIT surveillance group and the no follow-up surveillance group | Same |

Abbreviations: CRC, colorectal cancer; FIT, fecal immunochemical test; RWD, real-world data.

**Table B.** Specification and emulation of a target trial for CRC follow-up with direct colonoscopy and non-follow-up surveillance strategies.

| **Component** | **Target trial** | **Emulated trial using RWD** |
| --- | --- | --- |
| Design | Open-label two-parallelarm superlority randomized trial | Cohort study |
| Aim | Estimate the effect of receiving follow-up direct colonoscopy on the long-term CRC risk in individuals who have undergone adenoma removal | Same |
| Inclusion | Individuals with adenoma removal at baseline colonoscopy | Same |
| Exclusion | CRC detected by colonoscopy | Same |
| Treatment strategies | 1. Follow-up direct colonoscopy surveillance within 8.9 years after adenoma removal  2. Refusing follow-up surveillance within 8.9 years after adenoma removal | Same |
| Treatment assignment | Patients are randomly assigned to either strategy | Patients are non-randomly assigned to a follow-up surveillance strategy. Randomization is emulated via cloning of patients in both arms |
| Treatment implementation | None | 8.9 years grace period |
| Outcome | Incident CRC after surveillance strategies assignment | Same |
| Type of outcome | Time of CRC development | Same |
| Follow up | Follow up starts at adenoma removal, equivalent to surveillance assignment | Follow up starts at adenoma removal, which does not correspond to surveillance assignment |
| Censoring | Loss to follow up, administrative censoring, death | Loss to follow up, administrative censoring, death |
| Adjustment variables | Age, sex, adenoma grade at baseline colonoscopy, CRC family history in first-degree relatives, clinical symptoms, chronic appendicitis or cholecystitis, stressful life events | Same |
| Causal contrast andanalysis plan | Intention-to-treat and per-protocol effect analysis: A weighted Kaplan-Meier estimator and Cox regression were used to compute 14.4-year risk differences and hazard ratio | Per protocol effect only. Analysis in each arm of the cloned data, a weighted Kaplan Meier estimator and Cox regression were used to compute 14.4-year risk differences and hazard ratio |
| Estimands | Differences in incident CRC rate and hazard ratio between the direct colonoscopy surveillance group and the no follow-up surveillance group | Same |

Abbreviations: CRC, colorectal cancer; FIT, fecal immunochemical test; RWD, real-world data.

**Table C.** Demographics of the population at the second round of CRC screening.

| **Characteristic** | **Total**  **(N = 2441)** | **Direct colonoscopy  (N = 987)** | **FIT surveillance ^a^ (N = 1,194)** | **P value** |
| --- | --- | --- | --- | --- |
| **Age (mean, SD [year])** | 62.87 (7.09) | 62.42 (7.14) | 63.07 (7.06) | 0.033 |
| **Sex, n (%)** |  |  |  | 0.930 |
| Males | 1489 (61.0) | 607 (61.5) | 731 (61.2) |  |
| Female | 952 (39.0) | 380 (38.5) | 463 (38.8) |  |
| **Advanced adenoma, n (%) ^b^** |  |  |  | 0.459 |
| No | 1974 (80.9) | 806 (81.7) | 959 (80.3) |  |
| Yes | 467 (19.1) | 181 (18.3) | 235 (19.7) |  |
| **CRC family history in first-degree relatives, n (%)** |  |  |  | 0.286 |
| No | 2158 (88.4) | 877 (88.9) | 1042 (87.3) |  |
| Yes | 283 (11.6) | 110 (11.1) | 152 (12.7) |  |
| **Clinical symptom, n (%) ^c^** |  |  |  | <0.001 |
| No | 2268 (92.9) | 944 (95.6) | 1088 (91.1) |  |
| Yes | 173 (7.1) | 43 (4.4) | 106 (8.9) |  |
| **Chronic appendicitis or cholecystitis, n (%)** |  |  |  | 0.054 |
| No | 1652 (67.7) | 697 (70.6) | 796 (66.7) |  |
| Yes | 789 (32.3) | 290 (29.4) | 398 (33.3) |  |
| **Stressful life events, n (%)** |  |  |  | 0.188 |
| No | 2435 (99.8) | 987 (100.0) | 1190 (99.7) |  |
| Yes | 6 (0.2) | 0 (0.0) | 4 (0.3) |  |

^a^, FIT surveillance group refers to adherence to the complete process of FIT surveillance.

^b^, Advanced adenoma, any conventional adenoma ≥10 mm in size, or with advanced histology (high-grade dysplasia or villous/tubulovillous histology).

^c^, Clinical symptom was defined as individuals experiencing diarrhea, constipation, or bloody stool with mucus.

Abbreviations: CRC, colorectal cancer; SD, standard deviation; FIT, fecal immunochemical test.

**Table D.** The risk factors associated with advanced adenoma recurrence among the population following the removal of adenoma after the first-round colonoscopy.

| **Variable** | **Negative colonoscopy**  **(N = 864)** | **Advanced adenoma**  **(N = 42)** | **P value** | **OR (95%CI)** | **P value** | **aOR (95%CI) ^a^** | **P value** |
| --- | --- | --- | --- | --- | --- | --- | --- |
| **Age, n (%)** |  |  |  |  |  |  |  |
| < 60 | 312 (36.5) | 14 (33.3) | 0.797 | Ref |  |  |  |
| ≥ 60 | 542 (63.5) | 28 (66.7) |  | 1.15 (0.60, 2.22) | 0.674 | 1.92 (0.77, 4.78) | 0.159 |
| **Sex, n (%)** |  |  |  |  |  |  |  |
| Female | 380 (44.0) | 10 (23.8) | 0.016 | Ref |  |  |  |
| Males | 484 (56.0) | 32 (76.2) |  | 2.51 (1.22, 5.18) | 0.013 | 0.96 (0.27, 3.41) | 0.951 |
| **Alcohol consumption ^b^, n (%)** |  |  |  |  |  |  |  |
| > 3 times/week | 118 (13.7) | 11 (26.2) | 0.173 | Ref |  |  |  |
| 1-2 times/week | 10 (1.2) | 1 (2.4) |  | 1.07 (0.13, 9.18) | 0.949 |  |  |
| 1-2 times/month | 252 (29.2) | 12 (28.6) |  | 0.51 (0.22, 1.19) | 0.12 |  |  |
| Never | 110 (12.7) | 5 (11.9) |  | 0.49 (0.16, 1.45) | 0.196 |  |  |
| Unkown | 374 (43.3) | 13 (31.0) |  |  |  |  |  |
| **Aspirin use, n (%)** |  |  |  |  |  |  |  |
| Yes | 29 (8.2) | 1 (6.2) | 1.000 | Ref |  |  |  |
| No | 324 (91.8) | 15 (93.8) |  | 1.34 (0.17, 10.53) | 0.779 |  |  |
| **Smoking ^c^, n (%)** |  |  |  |  |  |  |  |
| No | 528 (61.1) | 18 (42.9) | 0.066 | Ref |  |  |  |
| Current | 216 (25.0) | 18 (42.9) |  | 2.44 (1.25, 4.79) | 0.009 | 3.72 (1.19, 11.60) | 0.024 |
| Former | 18 (2.1) | 1 (2.4) |  | 1.63 (0.21, 12.89) | 0.643 | 2.19 (0.22, 21.83) | 0.502 |
| Unkown | 102 (11.8) | 5 (11.9) |  |  |  |  |  |
| **BMI ^d^, n (%)** |  |  |  |  |  |  |  |
| Normal | 286 (33.1) | 14 (33.3) | 0.016 | Ref |  |  |  |
| Underweight | 24 (2.8) | 0 (0.0) |  | - | 0.986 | - | - |
| Overweight | 170 (19.7) | 8 (19.0) |  | 0.96 (0.40, 2.34) | 0.931 | 0.90 (0.36, 2.25) | 0.819 |
| Obese | 42 (4.9) | 7 (16.7) |  | 3.40 (1.30, 8.92) | 0.013 | 3.21 (1.17, 8.80) | 0.023 |
| Unkown | 342 (39.6) | 13 (31.0) |  |  |  |  |  |
| **Colonoscopy interval ^e^, n (%)** |  |  |  |  |  |  |  |
| < 6 years | 501 (58.0) | 25 (59.5) | 0.970 | Ref |  |  |  |
| ≥ 6 years | 363 (42.0) | 17 (40.5) |  | 0.94 (0.50, 1.76) | 0.844 |  |  |
| **Advanced adenoma at first-round colonoscopy ^f^, n (%)** |  |  |  |  |  |  |  |
| No | 688 (79.6) | 20 (47.6) | <0.001 | Ref |  |  |  |
| Yes | 176 (20.4) | 22 (52.4) |  | 4.30 (2.30, 8.06) | <0.001 | 3.30 (1.41, 7.69) | 0.006 |

^a^, Variables with missing data were excluded from the analysis.

^b^, Alcohol drinking was defind as consuming ≥ 100 grams of any alcohol per week over the past 6 months.

^c^, Former and current smoking were defined as quitting smoking for more than 6 months before colonoscopy and consuming at least one cigarette per day for more than one year or consuming over 300 cigarettes within 3 months, respectively.

^d^, BMI was classified according to the the guideline for Chinese adults: < 18.5 kg/m^2^ (underweight), 18.5 kg/m^2^ ≤ BMI < 24 kg/m^2^ (normal), 24 kg/m^2^ ≤ BMI < 28 kg/m^2^ (overweight) and ≥ 28 kg/m^2^ (obese).

^e^, Colonoscopy interval refers to the time period between the first and second-round colonoscopy examinations.

^f^, Advanced adenoma, any conventional adenoma ≥10 mm in size, or with advanced histology (high-grade dysplasia or villous/tubulovillous histology).

Abbreviation: OR, odds ratio; aOR, adjusted odds ratio; CI, confidence interval; BMI, body mass index.
